# Tooth Bleaching of Discolorations Caused by Hydraulic Cements in Regenerative Endodontic Treatment: A 3-Year In Vitro Study

**DOI:** 10.3390/ma15217845

**Published:** 2022-11-07

**Authors:** Carmen Llena, Manuel Iglesias-Diaz, Paula Ciscar-Muñoz, Ana Belén Bataller-Martínez, María Melo, José Luis Sanz

**Affiliations:** Department of Stomatology, Faculty of Medicine and Dentistry, Universitat de València, 46010 Valencia, Spain

**Keywords:** regenerative endodontic treatment, bleaching, hydraulic cements, in vitro, endodontics

## Abstract

This study aimed to evaluate the color change caused by hydraulic cements after 3 years in vitro by simulating their use in regenerative endodontic treatment (RET) and to quantify the color change after external bleaching with 40% hydrogen peroxide at 1, 6, and 12 months of follow-up. Fifty teeth were treated simulating RET. Samples were distributed according to the hydraulic cement to be used (n = 10 per group): negative control (no cement), ProRoot-MTA, MM-MTA, TotalFill BC-RRM, or Biodentine. Three years after RET, two sessions of external bleaching with Opalescence Boost were performed. The color was measured in the cervical and incisal halves of the teeth at different time points: baseline, 3 years after performing RET, and after 1, 6, and 12 months after bleaching. The ΔL, Δa, and Δb were determined. A generalized linear model was used to compare color considering group and time. The ΔE_ab_ and the ΔE_00_ were calculated and the acceptability in color change was determined. Three years after RET, a reduction in lightness (negative ΔL values) was found in all groups. These values significantly increased 1 month after bleaching in all groups (*p* < 0.05) and reversed at 6 months. One month after bleaching, ΔE_00_ values (color difference tolerance (CIEDE2000)) ranged from very good (>3.6 ≤ 5.4) to excellent (>5.4). One year after bleaching, the color reverted to values similar to those found 3 years after RET. All groups became darker after RET. The color recovered and even improved compared with the baseline measurement after one month of bleaching but did not remain stable over time.

## 1. Introduction

Regenerative endodontic treatment (RET) appeared as an alternative to traditional apexification procedures for the treatment of necrotic immature permanent teeth, aiming to achieve apical periodontitis healing, continued tooth root development, increased fracture resistance, and improved survival [1]. The RET process consists of the removal of the infected necrotic tissue, chemical disinfection and minor or instrumentation of the root canal system, induction of bleeding into the root canal, and placement of a coronal barrier with regenerative potential over the previously formed blood clot [2].

As a disinfection procedure, the use of tri-antibiotic pastes, which included minocycline, was initially proposed. Due to the discoloration caused by minocycline, this component was removed, leaving the antibiotic paste formed by the combination of cefaclor, amoxicillin, or clindamycin. Recently, antibiotic pastes have been replaced by calcium hydroxide for intracanal disinfection [3].

Hydraulic cements are a subgroup of bioceramic materials that contain ceramic crystals, calcium silicate, calcium phosphate, hydroxyapatite, radiopacifiers, and other metal oxides in their composition. They are biocompatible materials with osteoinductive potential, antimicrobial activity, and positive influence on cell proliferation and differentiation. They are used in endodontic therapy as sealing cements, for the repair of perforations or resorptions, retrograde filling, as pulp cappers in vital pulp treatment, and as coronal barriers in RET [4].

Mineral trioxide aggregate (MTA) was introduced in the field of endodontics as the first hydraulic material based on calcium silicates for root repair. However, although it exhibits great regenerative potential, it causes tooth discoloration [5]. It has been suggested that the bismuth oxide, which is used as a radiopacifier, together with other oxides within its composition are the cause of this discoloration [6]. Also, the interaction of MTA with blood and collagen from dentin may contribute to discoloration [7,8]. This manifests as a grayish staining of the tooth [9,10]. Also, contact with irrigant solutions such as sodium hypochlorite, which form sodium bismuthate and bismuth subcarbonate, as well as contact with chlorhexidine contribute to discoloration caused by MTA [11]. 

Several authors have evaluated tooth discoloration in vitro by comparing different MTA-based materials and other calcium silicate-based hydraulic cements in RET in the presence and absence of blood, some of which conclude that the presence of blood did not influence tooth discoloration [12]. However, other authors found greater discoloration in the presence of blood [8,13]. In both circumstances, MTA caused greater discoloration than the tricalcium silicate-based cement Biodentine.

Tooth discoloration can negatively affect quality of life, especially when treating anterior teeth, and particularly because affected patients are usually young. Tooth whitening is a simple and conservative procedure that has been shown to be effective for the treatment of dental discolorations derived from RET. In a systematic review conducted in 2021 [14], a series of in vivo studies were evaluated. Antibiotic pastes were used as intracanal medication, and MTA or Portland cement was used as the coronal barriers. All the in vivo studies were single case reports or case series, in which bleaching was performed with hydrogen peroxide, carbamide peroxide, or sodium perborate placed inside the pulp chamber, with mixed results in terms of bleaching efficacy and duration over time. In a study comparing Biodentine with MTA-based materials, a significant color improvement was found in the Biodentine group immediately after bleaching [12].

Most of the studies available in the literature that evaluated the dental discoloration caused by hydraulic cements used in RET were carried out with short follow-up times [6]. Likewise, the color change was immediately evaluated after bleaching and scarce information is available in the medium and long term [14]. This justifies the present in vitro study, whose objectives were (1) to evaluate, using a spectrophotometer, the discoloration potential of different hydraulic cements simulating their use in RET, three years after their application; and (2) to quantify the color change after two sessions of external bleaching with 40% hydrogen peroxide and the color variation after one, six, and twelve months after bleaching.

## 2. Materials and Methods

For the present study, single-rooted human teeth extracted for periodontal reasons and with an extraction period of no more than one year were used. The study was approved by the Ethics Committee of the Universitat de València with reference number H152078413452. The donors signed a written informed consent form beforehand.

### 2.1. Sample Selection and Preparation

All the teeth were visualized under 10× magnification, and two radiographs were taken to confirm the radicular status. Teeth with caries, restorations, structural alterations (cracks, fractures, and fissures), developmental anomalies, calcifications, staining, structural color alterations, or previous root canal treatment were excluded. A sample of 50 intact teeth was obtained. The teeth were kept in 0.1% thymol for 48 h and externally cleaned of organic debris, stains, or calculus and then placed in a saline solution for preservation in individual Eppendorf tubes for each specimen.

A transversal cut of the root apex was made with a diamond disk 10 mm from the cementoenamel junction (CEJ) to simulate an open apex. Subsequently, an access cavity was opened using a diamond burr. Root canal shaping was then performed using RECIPROC® blue R50 files (VDW, Frankfurt am Main, Germany) (25 mm, 8% taper) with an endodontic motor (Reciproc Silver; VDW, Frankfurt am Main, Germany); following the clinical sequence recommended by the manufacturer. An amount of 2.5% Sodium hypochlorite (NaOCl) was used as an irrigant between each file. The final irrigation was performed with 2.5% NaOCl, followed by 0.9% saline, 17% EDTA, and saline again. Finally, the root canal was dried with paper points (#50). The apex was sealed with wax.

### 2.2. Regenerative Endodontic Treatment Simulation

To simulate RET, calcium hydroxide (Ultracal XS; Ultradent, South Jordan, UT, USA) was placed inside the root canal, sealing the access cavity with a cotton pellet and a temporary cement (Cavit; 3M Espe St Paul, MN, USA). Teeth were stored at 37 °C in a 100% humidity environment for 4 weeks. After the storage period, the temporary filling was removed, and the root canal was washed with 10 mL of saline. Finally, the canal was left filled with saline. At 6 mm from the CEJ, 1 mm of Teflon was placed, and 4 mm of the hydraulic material under observation was placed over it, according to the distribution shown in Table 1. Then, a humid cotton pellet and temporary cement (Cavit) were placed over it, and the teeth were kept for 48 h in an environment of 100% humidity at 37 °C. After this time, the temporary filling was removed and the access cavity was sealed with an A1 shade Luna composite resin (SDI, Victoria, Australia) preceded by 37% orthophosphoric acid etching and the application of a universal adhesive system (Zipbond Universal; SDI, Victoria, Australia). The teeth were preserved in individual Eppendorf tubes with a saline solution for the duration of the study, which was changed every month.

### 2.3. Tooth Whitening Process Simulation

Three years after the RET simulation procedure, the coronal surfaces of the tooth samples were polished with a prophylaxis brush and an abrasive paste. Subsequently, 40% hydrogen peroxide in gel format was applied (Opalescense Boost; Ultradent, Madrid, Spain) on the coronal surfaces of the tooth samples with a microbrush (three 20-min applications, removing the whitening agent between each application with a thorough rinsing with water). After one week, the application of the whitening agent was repeated following the same protocol.

### 2.4. Tooth Color Evaluation

To evaluate change in tooth color, a white silicone base was made for each group with the individual imprint of each sample, which allowed the teeth to always be placed in the same position. A 1-mm thick thermoplastic splint with two perforations was made for each tooth, one in the coronal-cervical portion and the other in the coronal-incisal portion, both 5 mm in diameter, with the aid of a circular scalpel. 

For the color measurement, a Vita Easyshade spectrophotometer (Germany) was used, which provides the chromatic coordinates of the color in the CIELab space (L, a*, b*). L stands for the amount of black or white in a color, a* stands for the amount of red or green in a color, and b* stands for the amount of yellow or blue. The device was calibrated after the evaluation of every 10 samples. Each measurement was repeated until the same measurement values were obtained twice.

The color measurement was performed before performing RET simulation (baseline), 3 years after RET simulation, and 1, 6, and 12 months after the bleaching procedure.

### 2.5. Statistical Analysis

**Figure 1:**
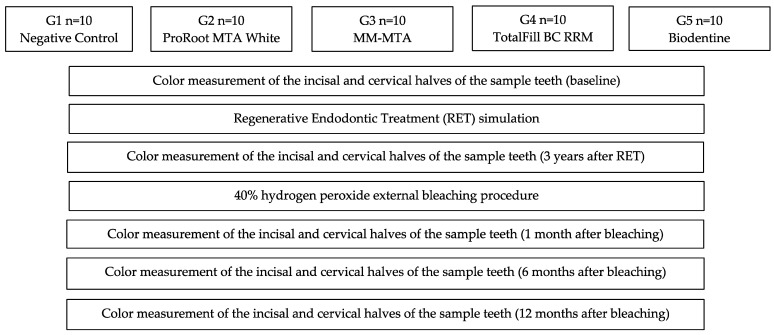
Study protocol flowchart.

The L a* b* values at each time of the study were recorded in an Excel sheet for analysis. The ΔL (difference in lightness values between two time points), Δa (difference in red or green values between two time points), and Δb (difference in yellow or blue values between two time points) were calculated for each group between the baseline values and the different times of the study, as schematized in Figure 1. The three variables were related by calculating the ΔE_ab_ (chromatic distance in the CIELab space between two time points), according to the formula:

ΔE_ab_ = ((L_a_ − L_b_)^2^ + (a_a_ − a_b_)^2^ + (b_a_ − b*_b_)^2^) ^½^

The ΔE00 (CIEDE2000) was also calculated, according to the formula:

ΔE_00_ = [(ΔL′/k_L_S_L_)^2^ + (ΔC′/k_c_S_c_)^2^ + (ΔH′/k_H_S_H_)^2^ + R_T_(ΔC′/k_c_S_c_)(ΔHv′/k_H_S_H_)] ^½^

The CIEDE2000 formula allows a better correlation between color difference and visual tolerance (perceptibility and acceptability) [15].

All calculations were performed for both the incisal and cervical halves of the teeth. The SPSS 28.0 statistical package (IBM, Chicago, IL, USA) was used. Two-way repeated measures ANOVA (analysis of variance) followed by Bonferroni posttest was performed to analyze the ΔL, Δa, Δb, ΔEab, and ΔE_00_, taking into account the treatment groups and evaluation periods. Differences were considered significant when *p* < 0.05.

## 3. Results

### 3.1. Inicisal Half

**Figure 2:**
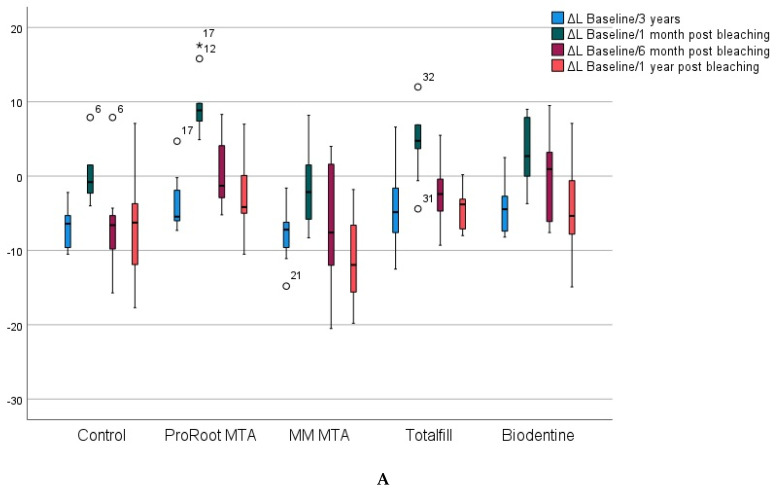

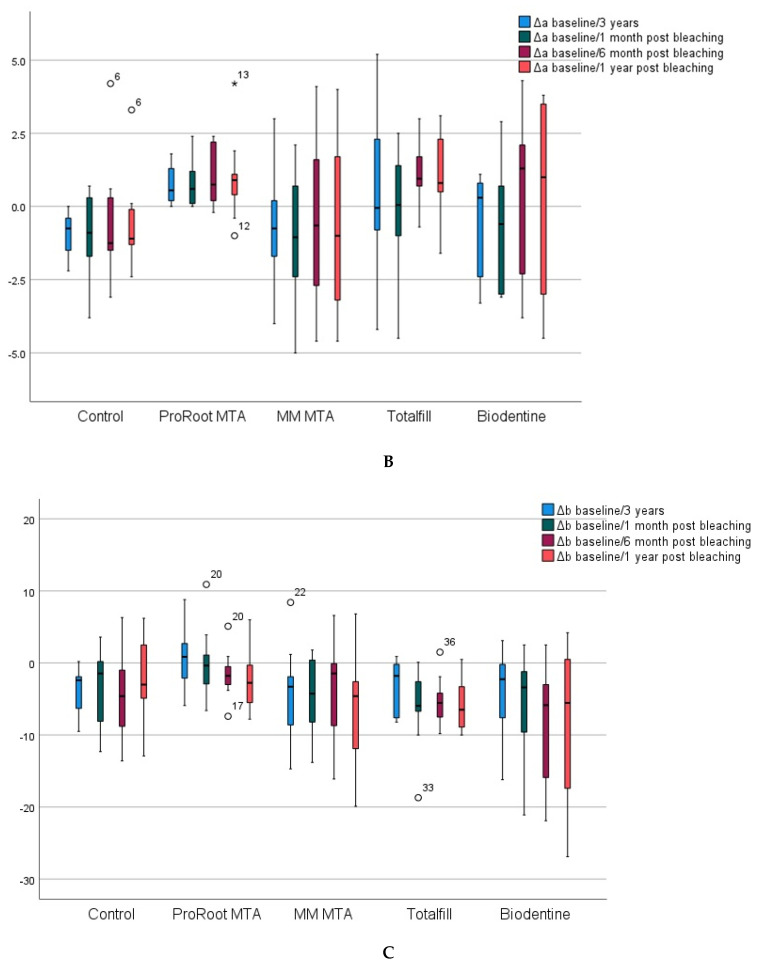
(**A**) Variations in ΔL (lightness) in the incisal halves. Boxplots showing the median values and IQR of the baseline and subsequent color measurements. * *p* < 0.05. (**B**) Variations in Δa (red–green distance) in the incisal halves. Boxplots showing the median values and IQR of the baseline and subsequent color measurements. * *p* < 0.05. (**C**) Variations in Δb (yellow–blue distance) in the incisal halves. Boxplots showing the median values and IQR of the baseline and subsequent color measurements. * *p* < 0.05.

In Figure 2A, a box plot is shown with the median and interquartile range (IQR) of the ΔL value between the baseline and each of the subsequent color measurements. In all groups, there was a reduction in lightness after the three-year follow-up with no significant differences between groups (*p* > 0.05). TotalFill and Biodentine groups showed the lowest reduction, around 4 points in both groups, while in the rest it was around 6.

One month after bleaching was performed, a significant increase in lightness was found in all groups (*p* < 0.05). In the ProRoot MTA group, the ΔL was significantly higher than all other groups (*p* < 0.05) at 9 units above the baseline value. Only the control group and the MM-MTA group did not reach a lightness value equal to or higher than the baseline value (the ΔL had negative values, which means that the lightness value after bleaching was lower than the baseline). 

Six months after bleaching, lightness values decreased in all groups, with a significantly higher ΔL in the ProRoot MTA group compared with the MM-MTA and the control group (*p* < 0.05). Twelve months after bleaching, the MM MTA group showed the greatest reduction in lightness, around 11 units, showing a significant difference compared with the ProRoot MTA group (*p* = 0.02). 

Within each group, lightness values were similar 3 years after RET simulation and 12 months after bleaching (*p* > 0.05), and significantly lower than baseline values in all groups (*p* < 0.05). Figure 2A shows that the ΔL values were negative in all groups, indicating that the value of L (lightness) was lower 12 months after bleaching compared with the baseline value.

Variations in Δa (red–green distance) showed no significant differences (*p* > 0.01) either between groups at the different times of the study, or within each group throughout the follow-up period (Figure 2B). MM-MTA and Biodentine were the groups that showed the greatest variability at all times of the study.

At all times of the study, a reduction of the b component (yellow-blue distance) was found, as can be seen in Figure 2C, in which all the boxes are in negative values. No significant differences were found between the groups at the different times of the study or within each group during the follow-up period (*p* > 0.05). The greatest variability was found in the MM MTA and Biodentine groups, especially at 6- and 12-months post bleaching.

Regarding the ΔE_00_ and ΔE_ab_, in all groups, three years after RET simulation, there was a darkening above the acceptable range (ΔE_00_ > 1.8 and ΔE_ab_ > 2.7). One month after bleaching, a very effective (ΔE_00_ > 3.6 and ΔE_ab_ > 5.4) to excellent (ΔE_00_ > 5.6 and ΔE_ab_ > 8.1) result was found in all groups primarily at the expense of an increase in lightness (Figure 2A). At the end of the follow-up period, all groups showed a color reversion, returning to ΔE_00_ values similar to those found three years after RET (ΔE_00_ in the last column of Table 2). From the lowest to the highest level of acceptability, the groups were ordered as follows: MM MTA< control < Biodentine < Totalfill < ProRoot.

### 3.2. Cervical Half

**Figure 3:**
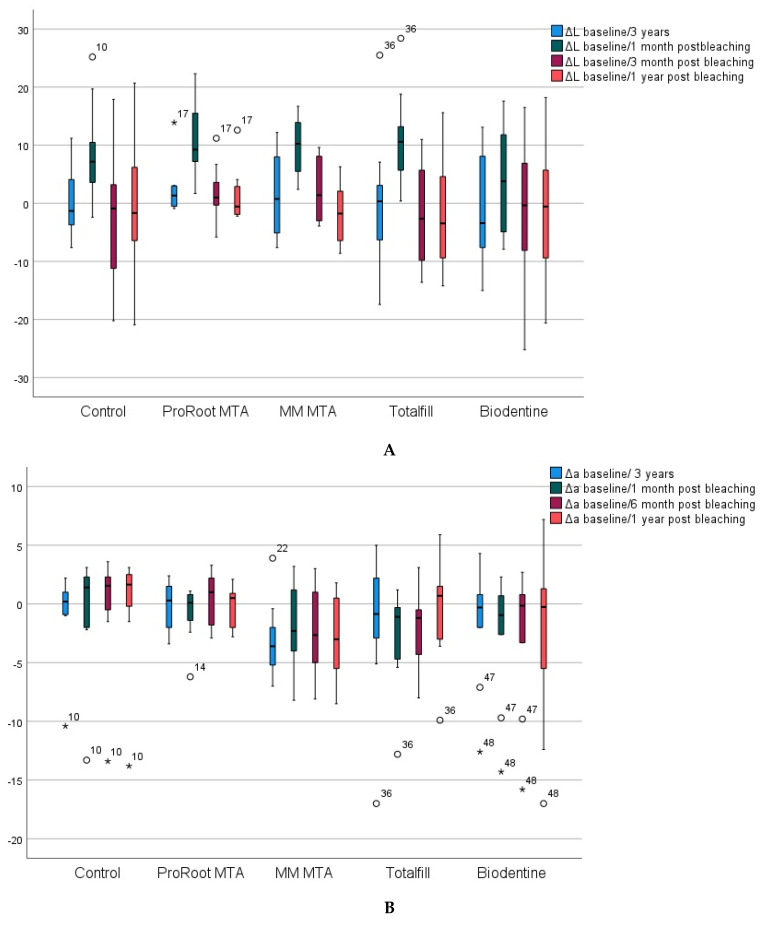

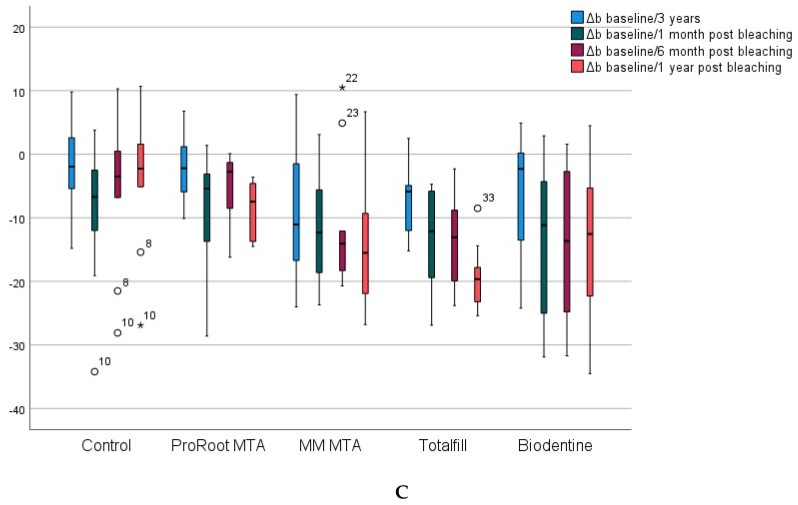
(**A**) Variations in ΔL (lightness) in the cervical halves. Boxplots showing the median values and IQR of the baseline and subsequent color measurements. * *p* < 0.05. (**B**) Variations in Δa (red–green distance) in the cervical halves. Boxplots showing the median values and IQR of the baseline and subsequent color measurements. * *p* < 0.05. (**C**) Variations in Δb (yellow–blue distance) in the cervical halves. Boxplots showing the median values and IQR of the baseline and subsequent color measurements. * *p* < 0.05.

In the cervical half, 3 years after RET simulation, lightness (ΔL) remained similar to baseline values in all groups (median ΔL close to 0). After bleaching, lightness increased significantly in all groups, compared with the baseline value and to the value 3 years after RET simulation (*p* < 0.05), although without significant differences between them (*p* > 0.05). 6 months after bleaching, values were similar in all groups to those obtained after RET simulation (*p* > 0.05) (Figure 3A).

Δa values (red–green distance) remained without significant differences between the different study groups and within each group at the different color measurement times (*p* > 0.05) (Figure 3B). 

The same was the case for the Δb values (blue–yellow distance), except for the TotalFill group, where the value of b* was significantly reduced at 12 months after bleaching compared with the value at three years after RET simulation (Figure 3C).

Using the ΔE_00_ and the ΔE_ab_ values, the level of acceptability in color change throughout the study was determined. In all groups, the ΔE_00_ values obtained were within the limits of very good or excellent acceptability 1 month after bleaching, fundamentally at the expense of an increase in lightness (Figure 3A). Six and twelve months after bleaching, ΔE_00_ values reverted. Comparing the baseline values before RET with the values 1 year after bleaching, from lowest to highest level of acceptability the groups were ordered as follows: MM-MTA < Control < Biodentine < ProRoot < TotalFill. ΔE_00_ and ΔE_ab_ values were similar to those obtained three years after RET simulation (Table 3). 

## 4. Discussion

Some authors report that the contact of the hydraulic materials used as a coronal barrier with the intracanal blood clot, which is crucial in the RET process, is an essential factor in tooth discoloration. This discoloration is often mainly attributed to the infiltration of iron and other hemoglobin derivatives into the dentin and coronal barrier materials [8,13]. Other studies, however, found no difference in the level of discoloration with or without contact with the blood of the hydraulic materials, especially when Biodentine was used [12]. In the present study, blood was not placed inside the root canal, since the intention was to exclusively evaluate the effect of the hydraulic materials. In addition, a 3-year follow-up was carried out, which would have caused a disintegration of the hematite that would have interfered with the objective of the study.

As described in the literature, the use of antibiotic pastes as disinfectants, especially if they contain tetracycline or synthetic derivatives of tetracycline such as minocycline or doxycycline, results in a higher level of discoloration than if bi-antibiotic pastes, pastes containing amoxicillin as a third antibiotic, or calcium hydroxide are used [10,11,16,17,18,19]. In the experimental model used in the present study, calcium hydroxide was used as a disinfectant.

According to a recent systematic review in the present field of study, hydrogen peroxide, carbamide peroxide at generally high concentrations (above 35%), or sodium perborate were used as bleaching agents in studies performing RET simulation. Bleaching products were intracoronally applied (internal bleaching) or on the coronal surface of the teeth (external bleaching). Follow-up times after bleaching ranged from 4 days to 3 weeks [14]. In the present study, we opted for an external bleaching technique with 40% hydrogen peroxide with two sessions of three applications each. We consider that if RET aims to form an intracanal inflow of mesenchymal stem cells and other progenitor cells capable of forming a mineralized tissue that reinforces the dentinal root walls, [20,21] placing peroxides in the pulp chamber of the tooth, even if there is a barrier material or a mineralized barrier has been formed, could have an undesirable cytotoxic effect. Thus, we chose to test the effect of an external bleaching technique [22].

To our knowledge, there are no studies that have evaluated the long-term effect of hydraulic materials used as coronal barriers in RET on tooth color, nor is there any information available on Totalfill regarding its potential discoloration effect and response to tooth whitening procedures.

The outcome of tooth whitening in in vitro and in vivo studies with RET exhibits different levels of effectiveness. Akbulut et al. [14] compared short-term discoloration caused by ProRoot MTA, MM-MTA, and Biodentine. They found discoloration in all groups after the application of the materials, with no significant differences between them. After bleaching, the Biodentine group obtained significantly better results. The authors attributed this result to the fact that the particle size of Biodentine is smaller than the other materials, as well as its porosity and to the difference in the radiopacifier (zirconium oxide in Biodentine and bismuth oxide in the other two materials). In another study, the discoloration produced by ProRoot MTA and MM-MTA two years after their application was compared. Their response to intracoronal bleaching with sodium perborate with a 6-week follow-up was also compared. The authors found a discoloration mainly at the expense of a reduction in lightness and an increase in the b* component, with a slight improvement in color after bleaching at the expense of an increase in lightness and a reduction in the b* component [23]. The present study found a slight improvement in color at the expense of an increase in lightness and a reduction in the b* component.

In the present study, it was found that three years after RET simulation, there was a darkening of all teeth in both the cervical and incisal halves. After bleaching, all groups achieved a significant improvement in color that reverted after 6 months. These changes were mainly produced by variations in lightness. Only the MM-MTA group showed significantly lower levels of lightness in the incisal half than the rest of the groups at the end of the follow-up period. The b* component (yellow–blue distance) showed a reduction in both the cervical and coronal halves, showing a shift towards blue. This may be due to the removal of the dentin needed to access the root canals and to the effect of the composite material used for the restoration, which was an A1 shade composite. The trend of negative Δb remained similar after bleaching and throughout the follow-up period, with no significant differences between the groups. The red–green distance (a* component) was reduced in all groups, except in the ProRoot MTA group, which showed no significant changes within each group throughout the study. Therefore, it should be noted that it is the lightness component that has shown the greatest influence on the color changes described in this study.

The combination of the three parameters was obtained by calculating the ΔEab [24]. The ΔE_00_ was also used, which incorporates the chrome and hue parameters proposed by Munsell and is more precise for determining tolerance to color changes [15]. However, both parameters can establish the ranges of perceptibility and acceptability in color change. However, they have the limitation of having no sign, i.e., the absolute value does not guide us towards an improvement or worsening of the color; therefore, to determine the directionality, they must be evaluated according to the change in their components. In the present study, discoloration could be seen in all groups above the acceptable range; three years after the regenerative therapy procedure (ΔE00 > 1.8 and ΔEab > 2.7), however, all groups experienced an improvement in color after bleaching in the very effective or excellent range (ΔE00 > 3.6 and ΔEab > 5.4). This improvement was not maintained over time, as can be seen in Table 1, the ΔE_00_ and ΔE_ab_ values decreased at 6 and 12 months after bleaching, returning to values similar to those found after RET simulation. Altogether, this indicates that bleaching was effective in all groups, but the results were not maintained in the medium or long term. These results agree with those obtained in the literature [1]. The results of the present study indicate that bleaching was effective in all groups, but the results were not maintained in the medium and long term.

## 5. Conclusions

Within the limitations of the present in vitro study, it can be concluded that discoloration was found in all groups, regardless of the hydraulic material used, and with no differences with regard to the control group after three years of RET simulation. The color improved after bleaching and was maintained in the short term but reverted in the medium and long term. The lightness component had the greatest influence on both discoloration and response to bleaching. Only the MM-MTA group showed significantly lower lightness values than the other groups at the end of the follow-up period in the incisal half.

## Figures and Tables

**Table 1:** Study groups and their composition and characteristics.

		Composition	Application
G1	Control negativo	No cement. Cotton pellet.	-
G2	ProRoot MTA White (Dentsply Maillefer, Ballaigues, Switzerland)	Powder: bismuth oxide, tricalcium silicate, dicalcium silicate, calcium dialuminate, and calcium sulfate dehydrated. Liquid: distilled water	Manually mixed powder/liquid (1:3 ratio)
G3	MM-MTA (Micro-Mega sur MedicalExpo, BESANCON Cedex, France).	Powder: tricalcium silicate, dicalcium silicate, tricalcium aluminate, bismuth oxide, calcium sulfate dehydrate, and magnesium oxide. Liquid: calcium carbonate	Automixed
G4	TotalFill BC RRM (FKG Dentaire, La Chaux-de-Fonds, Switzerland).	Paste: Calcium silicate, zirconium oxide, tantalum oxide, calcium phosphate monobasic, and fillers	Pre-mixed material and ready to apply
G5	Biodentine (Septodont, Saint-Maur-des-Fossés, France).	Powder: tricalcium silicate dicalcium silicate, calcium carbonate, iron oxide, and zirconium oxide. Liquid: Water, calcium chloride, and soluble polymer (polycarboxylate).	Automixed

**Table 2:** Variations in incisal ΔE values.

	Baseline/3 Years	3 Years/1 Month Post Bleaching	3 Years/6 Months Post Bleaching	3 Years/1 Year Post Bleaching	Baseline/1 Year Post Bleaching
	ΔE_00_	ΔE_ab_	ΔE_00_	ΔE_ab_	ΔE_00_
Control	4.23	7.73	4.57	6.82	4.63
ProRoot MTA	2.70	4.03	8.48	9.44	3.03
MM MTA	5.40	8.63	4.13	6.14	4.38
TotalFill	3.16	5.20	5.73	7.03	2.77

**Table 3:** Variations in cervical ΔE values.

	Baseline/3 Years	3 Years/1 Month Post Bleaching	3 Years/6 Months Post Bleaching	3 Years/1 Year Post Bleaching	Baseline/1 Year Post Bleaching
	ΔE_00_	ΔE_ab_	ΔE_00_	ΔE_ab_	ΔE_00_
Control	4.23	7.73	4.57	6.82	4.63
ProRoot MTA	2.70	4.03	8.48	9.44	3.03
MM MTA	5.40	8.63	4.13	6.14	4.38
TotalFill	3.16	5.20	5.73	7.03	2.77

## Data Availability

The data presented in this study are available on request from the corresponding author.
